# Assessment of vitamin D levels in patients with alopecia areata: a case-control study in a Tertiary Care Hospital in North India

**DOI:** 10.1080/07853890.2026.2662780

**Published:** 2026-04-26

**Authors:** Shivam Garg, K. Harsha Vardhan, Shitij Goel, Radha Rathi

**Affiliations:** Department of Dermatology, Venereology, and Leprosy School of Medical Sciences & Research, Sharda University Greater Noida, Uttar Pradesh, India

**Keywords:** Alopecia areata, vitamin D, 25-hydroxyvitamin D, case-control study, SALT score

## Abstract

**Introduction:**

Alopecia Areata (AA) is a T-cell-mediated autoimmune disorder. Vitamin D acts as an immunomodulator potentially relevant to pathogenesis. Since data from North India remains limited, this study aimed to assess serum 25-hydroxyvitamin D [25(OH)D] levels in AA patients compared to matched controls and evaluate correlations with disease severity.

**Methods:**

This hospital-based case-control study included 44 AA patients and 44 matched healthy controls. Disease severity was assessed using the Severity of Alopecia Tool (SALT) score. Serum 25(OH)D was measured using Chemiluminescence Immunoassay (CLIA).

**Results:**

Median serum 25- hydroxyvitamin D was significantly lower in cases (17.10 ng/mL) versus controls (22.60 ng/mL; *p* = 0.015). Deficiency (<20 ng/mL) was observed in 54.55% of cases versus 38.64% of controls. Vitamin D deficiency increased the odds of AA by 3.22 times (OR: 3.22; 95% CI: 1.18–8.79; *p* = 0.022). Stratified analysis revealed a significant difference only among urban residents (*p* = 0.041). No significant correlation was found between Vitamin D levels and SALT scores (*p* = 0.442).

**Conclusions:**

Serum 25- hydroxyvitamin D deficiency is significantly associated with Alopecia Areata in this North Indian cohort, particularly among urban residents. Screening for deficiency may be a valuable addition to management.

## Introduction

Alopecia Areata (AA) is a prevalent, chronic autoimmune disorder that manifests as the sudden onset of non-scarring hair loss in patchy patterns across hair-bearing regions. It demonstrates broad clinical heterogeneity, ranging from solitary discrete patches to total scalp alopecia (Alopecia Totalis) or complete body hair loss (Alopecia Universalis). While it affects approximately 2% of the global population with equal sex distribution, the peak incidence typically occurs during the second and third decades of life [1]. The disease’s unpredictability, combined with its profound cosmetic impact, frequently precipitates substantial psychological sequelae, including anxiety and depression, markedly diminishing the quality of life [2].

The pathogenesis of AA is complex but centres on the collapse of the relative immune privilege of the anagen hair follicle. Under normal physiological conditions, hair follicles are protected from immune surveillance. However, in AA, immune recognition of follicular autoantigens triggers an autoreactive cytotoxic T-cell (predominantly CD8+) assault on the follicle bulbs. This inflammation abruptly terminates the anagen phase, transitioning follicles into catagen and telogen phases, resulting in rapid shedding [3,4].

In recent years, the role of micronutrients in immunomodulation has garnered significant scientific interest. serum 25-hydroxyvitamin D [25(OH)D] has emerged as a key player, transcending its classical role in calcium and bone homeostasis. It functions as a secosteroid hormone, with its bioactive metabolite 1,25-dihydroxyvitamin D3 acting through the nuclear vitamin D Receptor (VDR). VDRs are distributed widely across immune cells, including T and B-lymphocytes, macrophages, and dendritic cells [5]. Biologically, vitamin D exerts potent immunomodulatory effects by suppressing the expansion of T-helper 1 (Th1) cells – critical effectors in organ-specific autoimmunity – and curtailing pro-inflammatory mediators such as IFN- γ and IL-2. Concurrently, it enhances the differentiation of regulatory T-cells (Tregs), which are crucial for maintaining self-tolerance and preventing autoimmunity [6].

Since AA is a T-cell-mediated autoimmune condition, a biological basis exists for suspecting Serum 25-hydroxyvitamin D’s involvement in its pathogenesis. However, global observational research has yielded heterogeneous findings. While studies from regions like Turkey and Europe have documented significantly reduced serum 25-hydroxyvitamin D levels in AA patients [7,8], large population-based studies from Scandinavia have reported null findings. Additionally, the prevalence of Serum 25-hydroxyvitamin D deficiency is a growing public health concern in the Indian subcontinent, potentially exacerbated by melanin-mediated synthesis reduction and cultural sun-avoidance practices [9].

Data specific to North India, and particularly the rapidly urbanizing region of Greater Noida, remains limited. The local population may be subject to unique environmental factors, such as atmospheric pollution and lifestyle shifts, that influence serum 25-hydroxyvitamin D status. Establishing an association in this demographic carries practical clinical significance, as serum 25-hydroxyvitamin D correction could serve as a safe, cost-effective adjuvant therapy. This study aimed to assess serum 25-hydroxyvitamin D [25(OH)D] levels in AA patients compared to matched controls in a tertiary care setting and to investigate correlations with disease severity.

## Materials and methods

### Study design and setting

This hospital-based, observational, case-control study was conducted at the Department of Dermatology, Venereology, and Leprosy, a tertiary care center in Greater Noida. The study was conducted in accordance with the ethical principles of the Declaration of Helsinki. The study spanned a period of 18 months, from May 2024 to November 2025, following approval from the Institutional Ethics Committee (Ref. No. SU/SMS&R/76-A/2024/137).

### Study population

The study enrolled a total of 88 participants, comprising 44 cases and 44 controls.

Cases: Included patients of either sex clinically diagnosed with any pattern of Alopecia Areata (patchy, totalis, universalis, or ophiasis) attending the Dermatology Outpatient Department (OPD).

- **Controls:** Included age- and sex-matched healthy individuals without any history or clinical evidence of hair loss disorders.

### Inclusion and exclusion criteria

Inclusion criteria for cases were a confirmed clinical diagnosis of AA and written informed consent. Exclusion criteria were strictly defined to eliminate confounding factors affecting serum 25-hydroxyvitamin D [25(OH)D] metabolism. Participants were excluded if they had scarring alopecia, other hair loss disorders, autoimmune/systemic diseases (e.g. thyroid disorders, rheumatoid arthritis), chronic kidney/liver disease, or malabsorption. Additionally, those who received systemic corticosteroids, immunosuppressants, phototherapy, or serum 25-hydroxyvitamin D [25(OH)D]/calcium supplementation in the preceding three months, as well as pregnant or lactating women, were excluded.

### Sample size calculation

The sample size was estimated based on the prevalence of alopecia areata in the Indian population (approx. 2%) and findings from previous studies. Using a standard normal variate (*Z*) of 1.96 for a 5% level of significance and a power of 80%, a sample size of 44 participants per group was calculated to detect a statistically significant difference, yielding a total sample size of 88.

### Data collection and clinical assessment

After obtaining informed consent, a detailed proforma was used to record demographic details (age, sex, occupation, residence) and relevant clinical history (duration of disease, onset, family history, nail changes).

#### Initial screening

A total of 71 patients of alopecia areata were screened during the study period.

#### Exclusions

27 patients were excluded based on strict criteria, including the presence of scarring alopecia, systemic diseases (e.g. thyroid disorders), or use of serum 25-hydroxyvitamin D [25(OH)D] supplements or systemic corticosteroids in the preceding three months.

#### Allocation

The remaining 44 participants were allocated as cases.

#### Matching strategy

44 controls were selected to match the case group’s demographic profile, specifically focusing on identical sex distribution (59.1% male) and comparable mean age (within ± 2 years) (Cases: 26.27 years; Controls: 25.52 years) to minimize confounding variables. (a flow chart is attached after figure legends).

Disease severity in AA patients was objectively quantified using the Severity of Alopecia Tool (SALT) score. The scalp was divided into four anatomical regions: vertex (40% scalp area), right profile (18%), left profile (18%), and posterior scalp (24%). The percentage of hair loss in each region was visually estimated, and the composite score was calculated using the formula:

SALT score=(0.40×vertex%)+(0.18×right%)+(0.18×left%)+(0.24×posterior%)

Based on the score, severity was categorized as Mild (<25%), Moderate (25–49%), or Severe (>50%).

### Biochemical analysis

Under aseptic conditions, 5 mL of venous blood was drawn from each participant into a plain vial (red top). Serum was separated and analyzed for 25-hydroxyvitamin D [25(OH)D] levels using Chemiluminescence Immunoassay (CLIA) in a NABL-accredited laboratory.

serum 25-hydroxyvitamin D [25(OH)D] status was classified according to the Endocrine Society’s clinical practice guidelines:

- **Deficiency:** <20 ng/mL
- **Insufficiency:** 21–29 ng/mL
- **Sufficiency:** ≥30 ng/mL
- **Toxicity:** >150 ng/mL

### Statistical analysis

Data were entered into Microsoft Excel and analyzed using IBM SPSS Statistics version 22.0. The normality of continuous variables was tested using the **Shapiro-Wilk test**. Variables conforming to a normal distribution are expressed as mean ± standard deviation, and those not conforming are expressed as median (interquartile range). Categorical variables were compared using the **Chi-square test**. Correlations were assessed using **Spearman’s rank correlation**. **Receiver Operating Characteristic (ROC)** curve analysis determined the diagnostic accuracy, and Odds Ratios (OR) were calculated to quantify risk. A P-value <0.05 was considered statistically significant.

## Results

### Demographic profile

**Figure 1.**
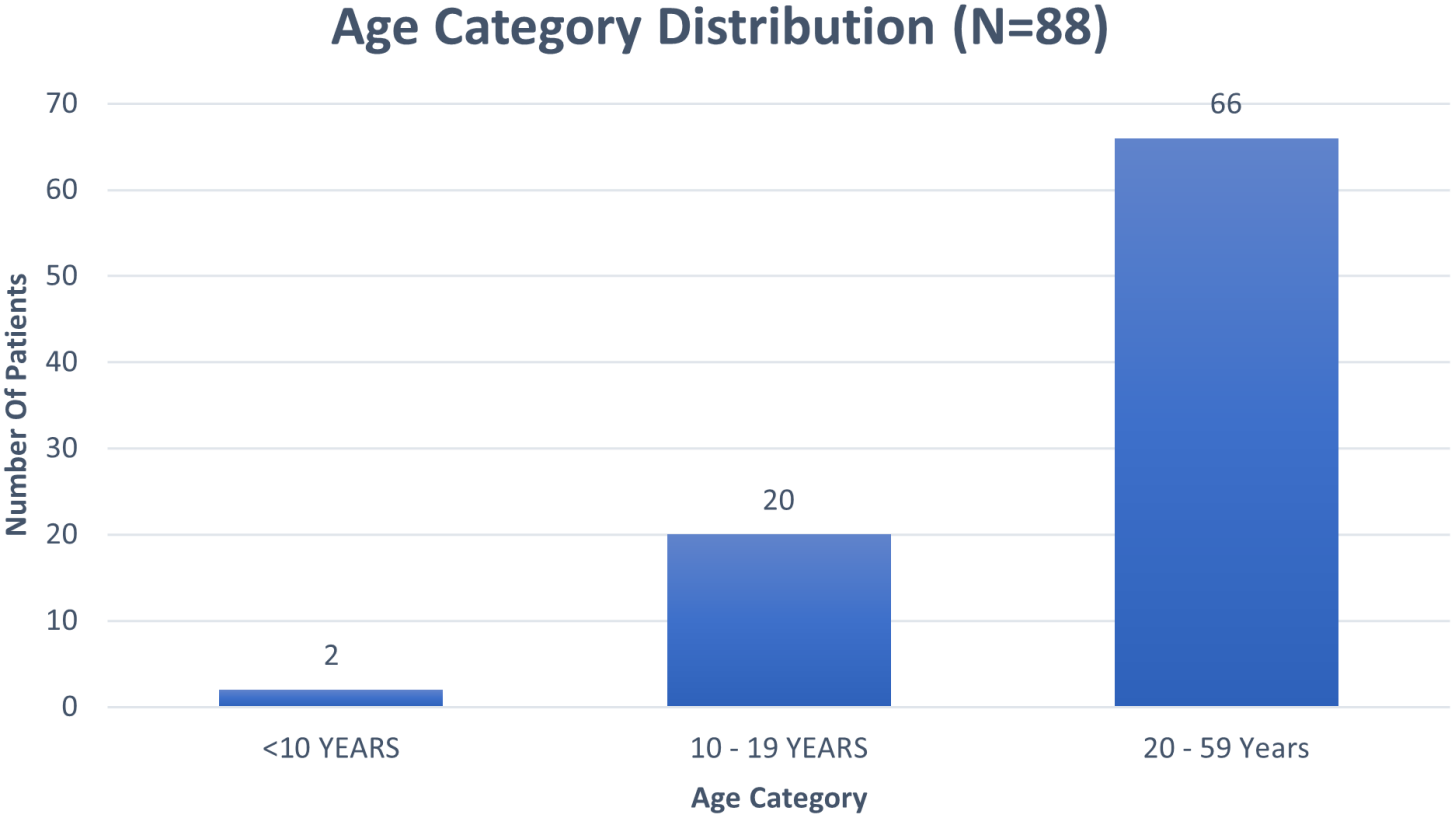
Age category distribution in the study population, showing the majority of participants in the 19–59 years age group.

**Figure 2.**
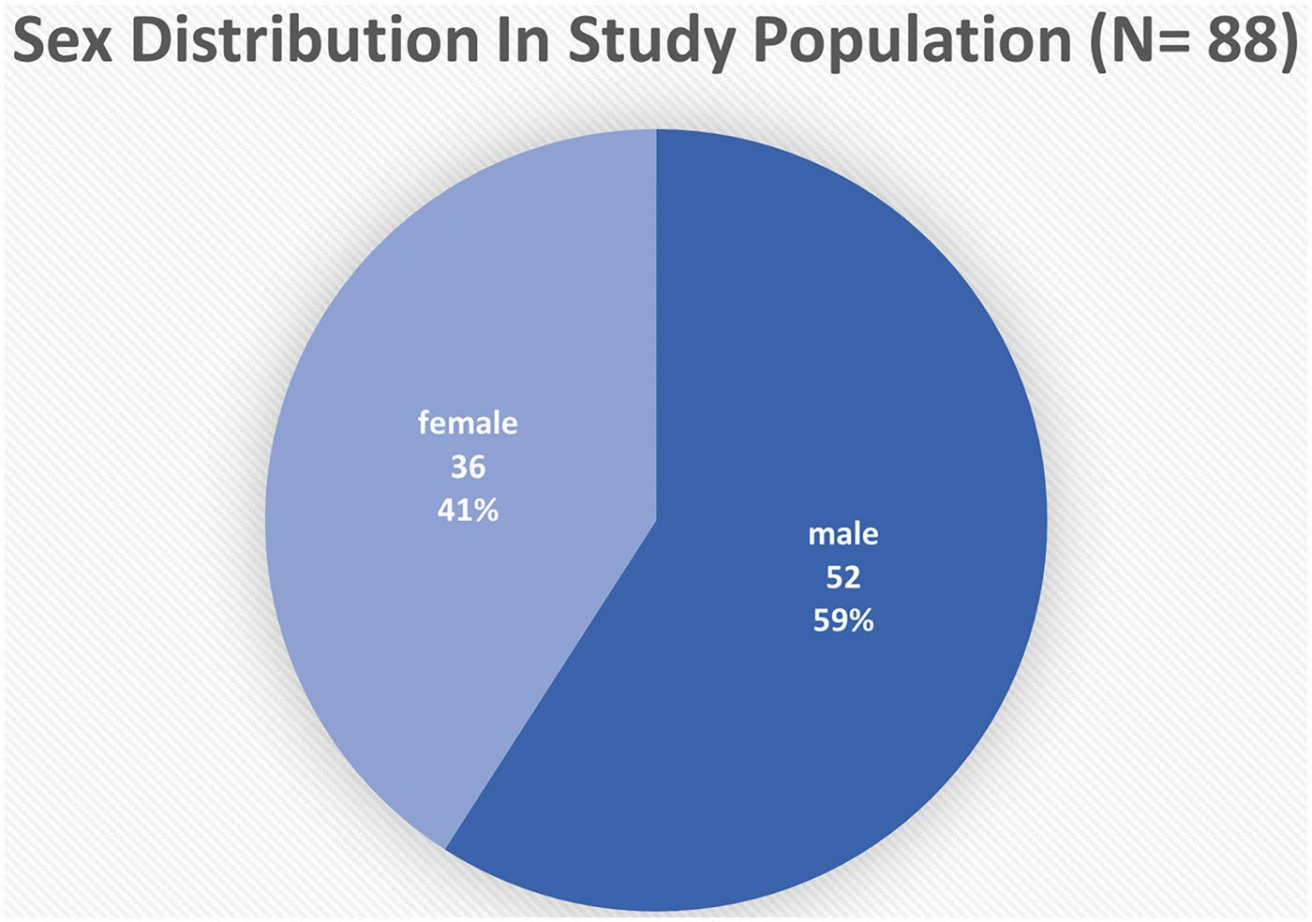
Sex distribution in the study population, showing a male predominance (59.1%).

The study cohort included 44 AA cases and 44 healthy controls, well-matched for demographic parameters. The mean age of the cases was *26.27 ± 9.59* years, while controls had a mean age of *25.52 ± 8.68* years (*p = 0.702*) (Table 1). The age distribution showed that the majority of participants (75%) fell within the 20–59 years age group, followed by adolescents (10–19 years) comprising 22.73% (Figure 1). The sex distribution was identical in both groups, with 26 males (59.09%) and 18 females (40.91%), reflecting a slight male predominance (Figure 2). Geographically, 57.95% of participants resided in urban areas, while 42.05% were from rural backgrounds.

**Table 1.** Comparison of age between cases and controls.

Age Group	Cases (*n* = 44)	Controls (*n* = 44)	P-value
<10 years	1 (2.27%)	1 (2.27%)	1.000
10–19 years	10 (22.73%)	10 (22.73%)	
20–59 years	33 (75.00%)	33 (75.00%)	
**Mean ± SD**	**26.27 ± 9.59**	**25.52 ± 8.68**	**0.702**

*SD: Standard Deviation; P-value calculated using Independent Samples t-test*.

### Clinical characteristics of AA cases

The clinical profile of the cases revealed that 70.45% experienced a sudden onset of hair loss. The scalp was the most frequently affected site (86.36%), followed by combined scalp and beard involvement (11.36%). All patients (100%) presented with the patchy pattern of alopecia. Regarding disease progression, 25% of cases reported a history of relapse. Nail changes, specifically pitting, were observed in 9.09% of cases. A family history of AA was present in only 9.09% of the study population.

Disease severity was predominantly mild; 93.18% of patients had a SALT score of 0–24%, while only 6.82% presented with moderate severity (SALT 25–49%). No cases of alopecia totalis or universalis were recruited.

### Serum 25-hydroxyvitamin D [25(OH)D] levels and status

**Figure 3.**
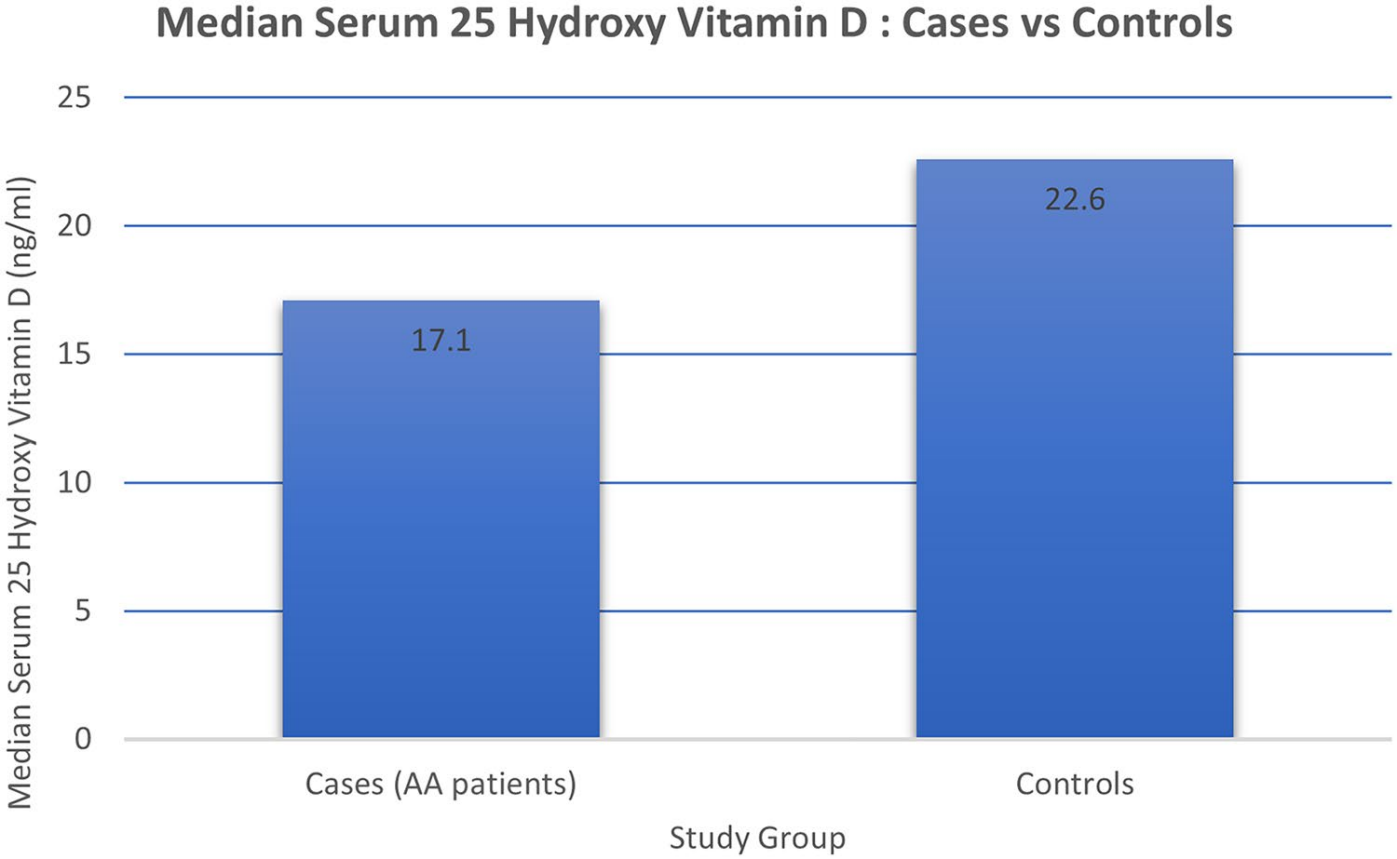
Comparison of median serum 25-hydroxyvitamin D [25(OH)D] levels (ng/mL) between cases (17.1 ng/mL) and controls (22.6 ng/mL).

The primary outcome analysis revealed significantly lower serum 25-hydroxyvitamin D [25(OH)D] levels in the AA group. The median serum 25(OH)D level was 17.10 ng/mL (IQR: 13.12–26.20) in cases, compared to 22.60 ng/mL (IQR: 17.65–31.82) in controls. This represented a median difference of 5.5 ng/mL, which was statistically significant (*p = 0.015*) (Figure 3).

**Figure 4.**
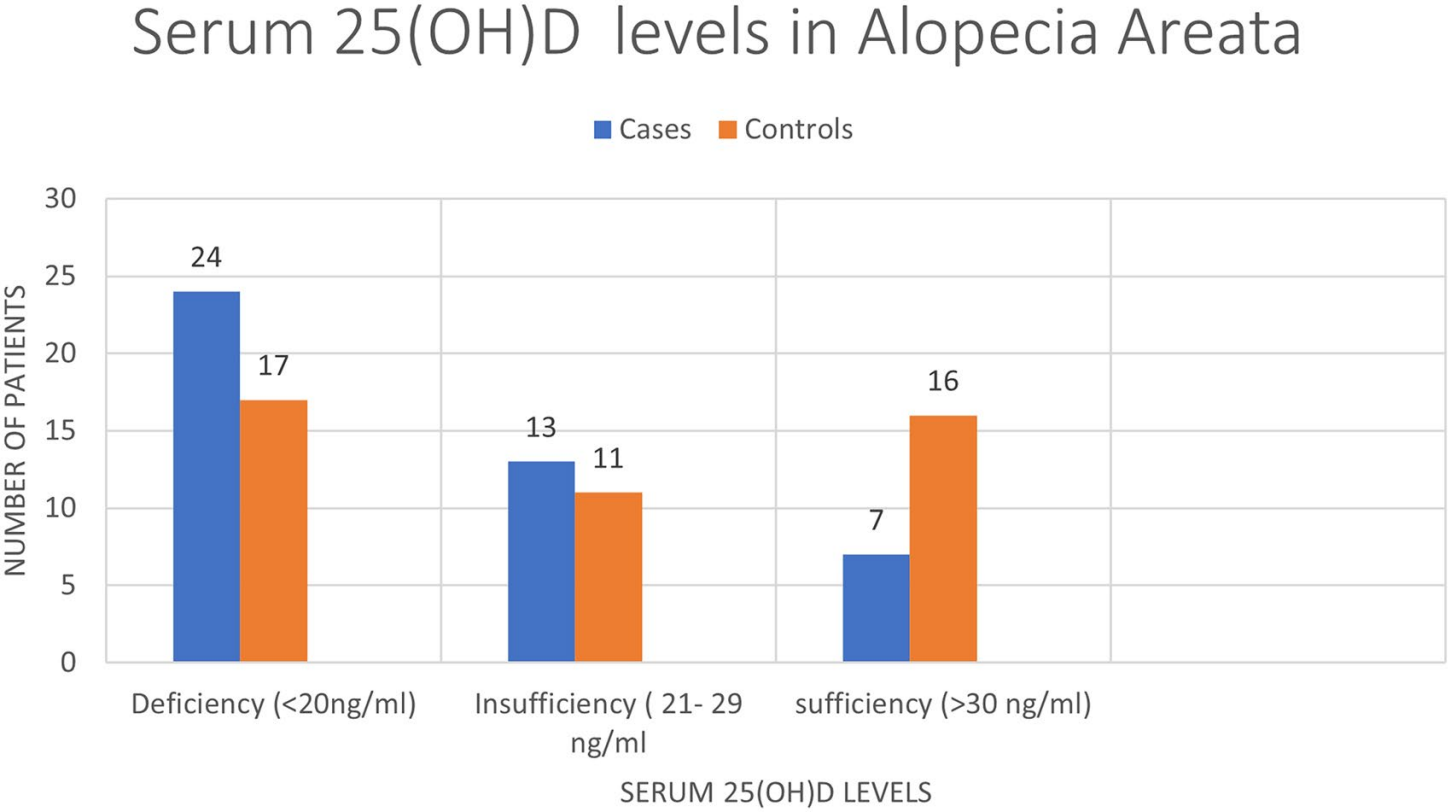
Distribution of serum 25-hydroxyvitamin D [25(OH)D] status categories comparing Alopecia Areata cases and healthy controls. Cases exhibited higher rates of deficiency (54.55%) compared to controls (38.64%).

When categorized by serum 25-hydroxyvitamin D [25(OH)D] status (Table 2, Figure 4):

**Table 2.** Comparison of serum 25-hydroxyvitamin D levels.

Serum 25-hydroxyvitamin D [25(OH)D] Status	Cases (*n* = 44)	Controls (*n* = 44)	P-value
Deficiency (<20 ng/mL)	24 (54.55%)	17 (38.64%)	0.087
Insufficiency (20-29.9 ng/mL)	13 (29.55%)	11 (25.00%)	
Sufficiency (≥30 ng/mL)	7 (15.91%)	16 (36.36%)	
**Median (IQR)**	**17.10 (13.12–26.20)**	**22.60 (17.65–31.82)**	**0.015** *

IQR: Interquartile Range.

* Statistically significant (*p* < 0.05) using Mann-Whitney U test.

- **Deficiency (<20 ng/mL):** Observed in **54.55%** of cases vs. **38.64%** of controls.
- **Insufficiency (21–29 ng/mL):** Observed in **29.55%** of cases vs. **25.00%** of controls.
- **Sufficiency (≥30 ng/mL):** Observed in only **15.91%** of cases vs. **36.36%** of controls.

The Odds Ratio (OR) analysis indicated that individuals with serum 25-hydroxyvitamin D [25(OH)D] deficiency had **3.22 times the odds** of having AA compared to those with sufficient levels (95% CI: 1.18–8.79; *p = 0.022*) (Table 4).

### Stratified analysis: urban vs. rural and gender

A novel finding emerged from the stratified analysis based on residence. The difference in median serum 25-hydroxyvitamin D [25(OH)D] levels was statistically significant among urban residents (*p = 0.041*) (Table 3), where urban cases had lower levels (17.20 ng/mL) than urban controls (22.50 ng/mL). In contrast, the difference among rural participants was not statistically significant (*p = 0.231*).

**Table 3.** Stratified analysis of median serum 25-hydroxyvitamin D [25(OH)D] levels (ng/mL) by residence.

Residence	Group	Median (IQR)	P-value
Urban	Cases (*n* = 26)	17.20 (13.95–24.30)	**0.041** *
	Controls (*n* = 25)	22.50 (18.80–30.10)	
Rural	Cases (*n* = 18)	16.65 (12.37–30.30)	0.231
	Controls (*n* = 19)	22.60 (14.10–33.60)	

* Significant association found only in urban population.

**Table 4.** Odds ratio for serum 25-hydroxyvitamin D [25(OH)D] deficiency.

Group	Deficient (<20 ng/mL)	Sufficient (≥30 ng/mL)	Odds Ratio (95% CI)	P-value
Cases	24	7	**3.22** (1.18–8.79)	0.022*
Controls	17	16	Reference	–

CI: Confidence Interval; Comparison excludes participants with Insufficiency (21–29 ng/mL).

**Figure 5.**
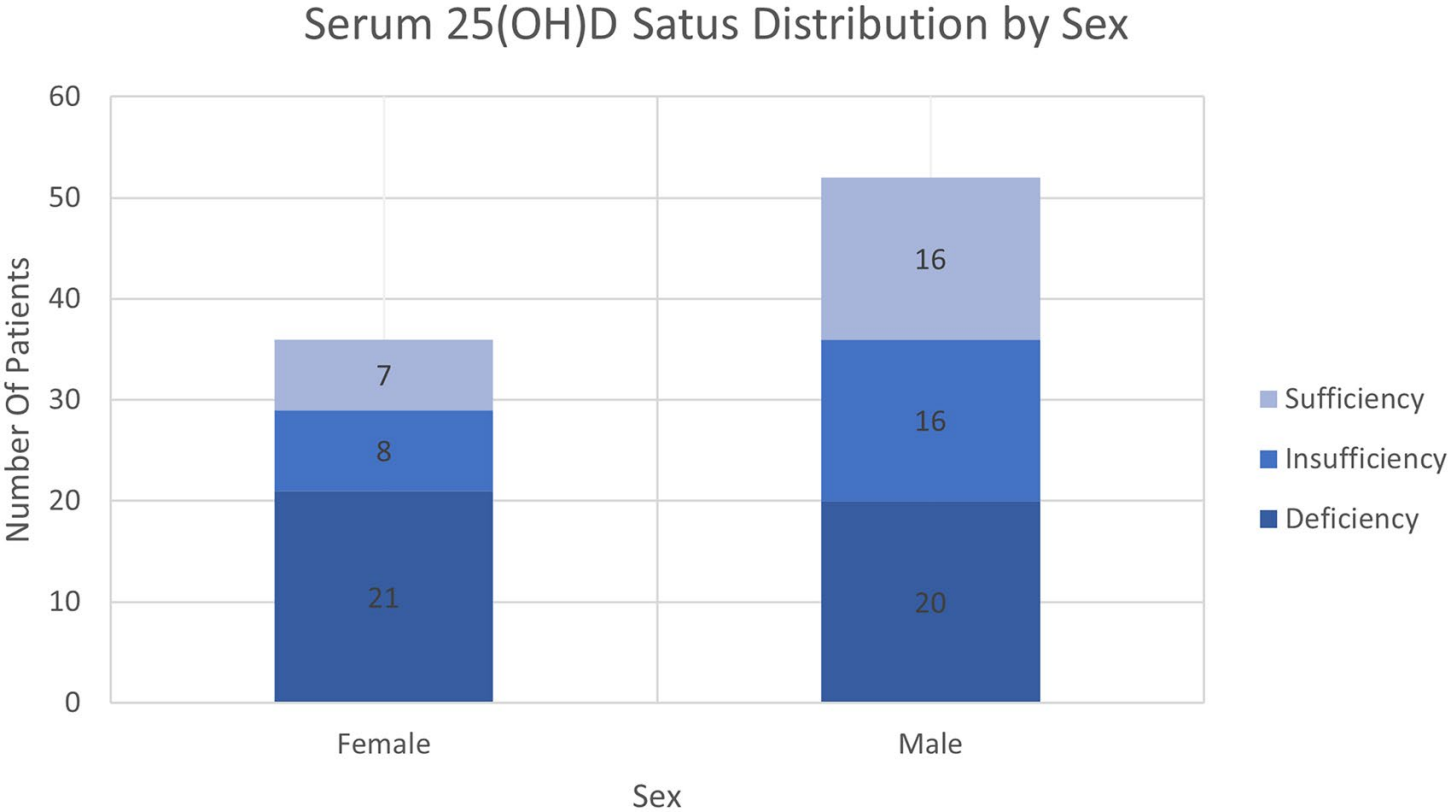
Stacked bar chart showing serum 25-hydroxyvitamin D [25(OH)D] status distribution by sex. Females exhibited higher deficiency rates compared to males.

Gender-stratified analysis showed that females generally had lower serum 25-hydroxyvitamin D [25(OH)D] levels than males (Figure 5). The deficiency rate was 58.3% in females compared to 38.5% in males.

### Correlation with severity and diagnostic accuracy

**Figure 6.**
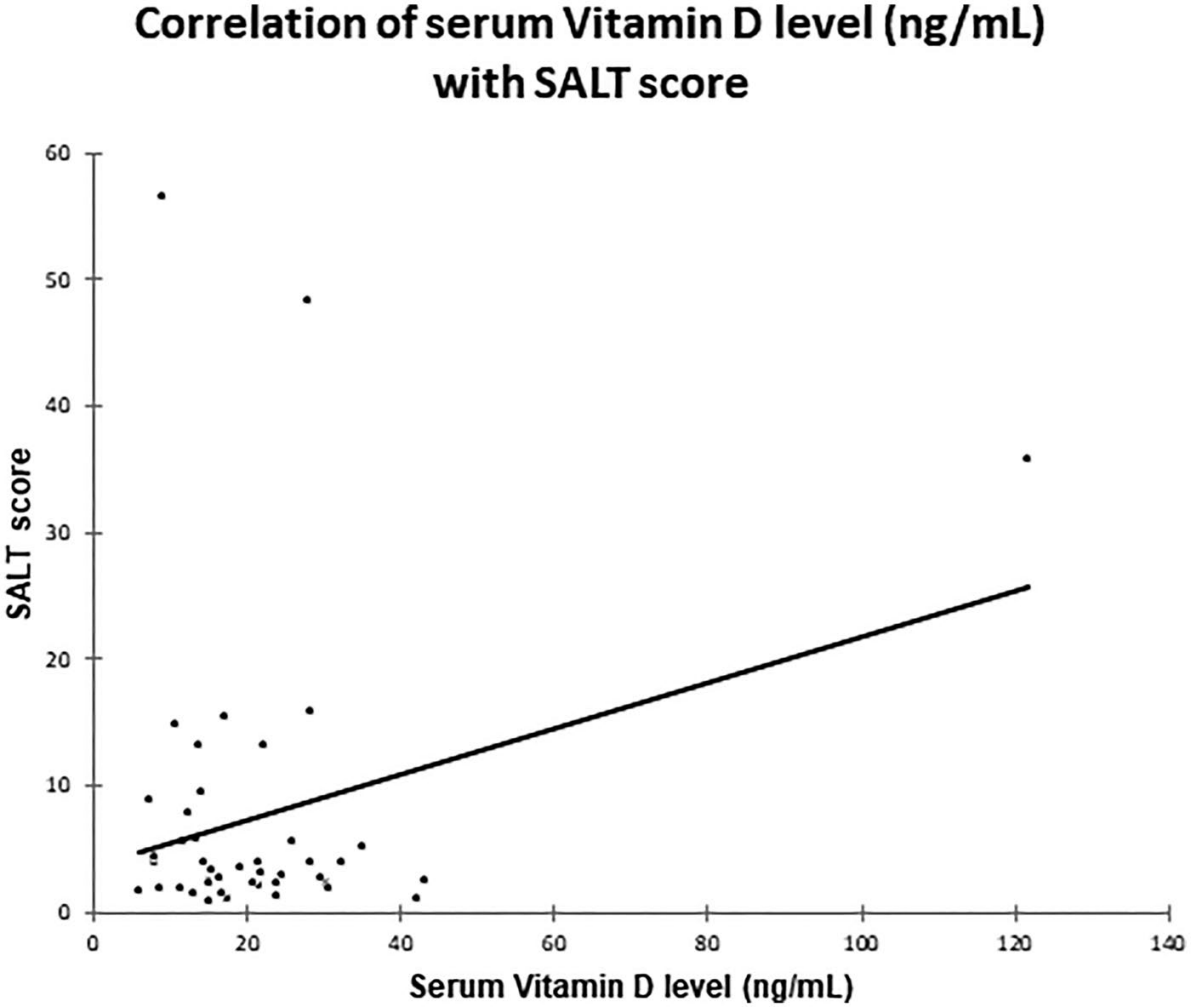
Correlation between serum 25-hydroxyvitamin D [25(OH)D] levels and SALT score in cases. No significant linear correlation was observed.

Spearman’s rank correlation analysis showed no significant linear correlation between serum 25-hydroxyvitamin D [25(OH)D] levels and disease severity (SALT score) (ρ = −0.063; *p = 0.442*) (Figure 6). Similarly, no significant associations were found between serum 25-hydroxyvitamin D [25(OH)D] levels and other clinical parameters such as disease duration, number of lesions, or nail changes.

**Figure 7.**
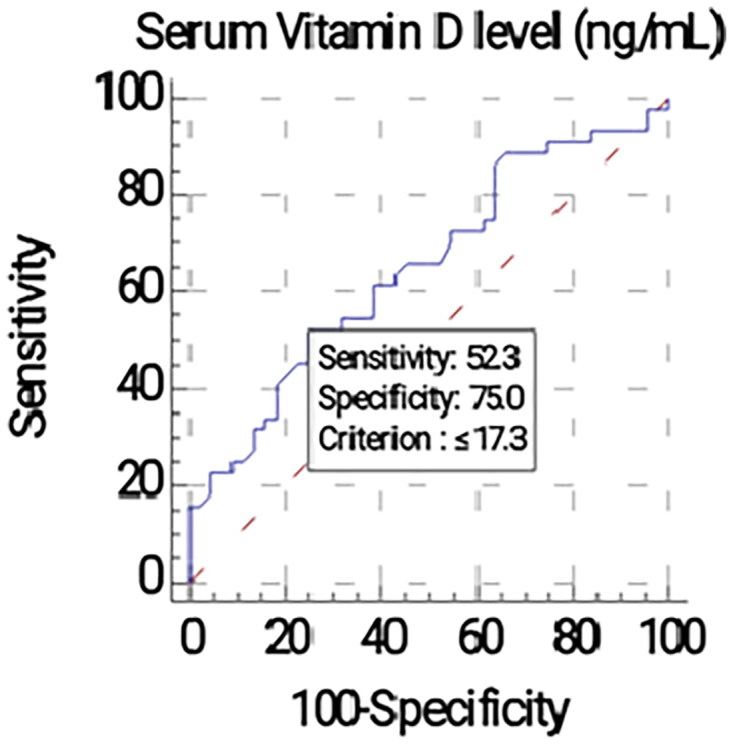
Receiver Operating Characteristic (ROC) curve for serum 25-hydroxyvitamin D [25(OH)D] levels as a predictor of Alopecia Areata. The optimal cut-off value was ≤17.3 ng/mL (AUC = 0.650).

ROC curve analysis demonstrated that serum 25-hydroxyvitamin D [25(OH)D] level is a significant predictor for AA (*p* = 0.0106). An optimal cut-off value of ≤ 17.3 ng/mL was identified, yielding a specificity of 75.00% and a sensitivity of 52.27% (AUC = 0.650), as shown in Figure 7.

## Discussion

This study provides compelling evidence of a significant association between lower serum 25-hydroxyvitamin D levels and Alopecia Areata in a North Indian population. Our cohort of AA patients exhibited a significantly lower median serum 25-hydroxyvitamin D [25(OH)D] level (17.10 ng/mL) compared to healthy controls (22.60 ng/mL), with a P-value of 0.015. This finding aligns with the growing body of literature supporting the immunomodulatory role of serum 25-hydroxyvitamin D [25(OH)D] in autoimmune diseases. The median difference of 5.5 ng/mL observed in our study is consistent with data from other Indian studies, such as Panda et al. from Eastern India, who reported a mean difference of 7.5 ng/mL [10]. It also corroborates the findings of a global meta-analysis by Lee et al. which demonstrated a standardized mean difference of −8.20 ng/mL in AA patients [8].

In contrast to our findings, a large population-based study from Denmark by Gade et al. found no significant association [11]. This discrepancy likely highlights the influence of geographic and population-specific factors. Scandinavian populations often have higher baseline serum 25-hydroxyvitamin D [25(OH)D] levels due to dietary fortification and distinct genetic factors, whereas the Indian population faces a high prevalence of endemic deficiency due to skin pigmentation and lifestyle factors. Our study reinforces that the serum 25-hydroxyvitamin D [25(OH)D] -AA relationship may be most clinically relevant in populations with high background deficiency.

A particularly novel and significant finding of this study is the **urban-rural differential**. We observed that the association between low serum 25-hydroxyvitamin D [25(OH)D] and AA was statistically significant only among urban residents (*p = 0.041*). This finding is biologically plausible and reflects the unique environmental challenges of rapidly urbanizing regions like Greater Noida. Urban populations are often exposed to higher levels of atmospheric pollution, which can scatter UVB radiation essential for cutaneous serum 25-hydroxyvitamin D [25(OH)D] synthesis. Additionally, urban lifestyles frequently involve indoor occupations and reduced sun exposure compared to rural agrarian lifestyles. This suggests that urban residents may face a ‘double hit’ of environmental serum 25-hydroxyvitamin D [25(OH)D] restriction and autoimmune susceptibility.

Despite the strong association with disease presence, we did not find a significant correlation between serum 25-hydroxyvitamin D [25(OH)D] levels and disease severity as measured by the SALT score (*p = 0.442*). This mirrors the findings of Panda et al. but contrasts with studies by Aksu Cerman et al. and Bhat et al. who reported inverse correlations with severity [7,12]. The most likely explanation for this divergence is the ‘floor effect’ in our study population. The vast majority of our cases (93.18%) presented with mild disease (SALT <25%), with no cases of alopecia totalis or universalis. In hospital-based settings where patients may present early for cosmetic concerns, the restricted severity range limits the statistical power to detect correlations. This suggests that serum 25-hydroxyvitamin D [25(OH)D] may be more critical for disease susceptibility (triggering the onset) rather than driving progression in early or mild cases.

The Odds Ratio of 3.22 (95% CI: 1.18–8.79) observed in our study indicates a substantial risk association, suggesting that serum 25-hydroxyvitamin D [25(OH)D] deficiency is a significant marker for AA. The ROC analysis further supports this, identifying a specific cut-off of ≤17.3 ng/mL with a high specificity of 75%. This threshold is clinically practical, falling close to the standard definition of deficiency (<20 ng/mL), and supports the utility of screening.

### Limitations

Includes a modest sample size, and the predominance of mild cases. Unmeasured confounders like BMI and quantitative sun exposure also limit generalizability. As the study population was derived from a single tertiary care center using specific inclusion and exclusion criteria, there is an inherent **risk of selection bias** that may influence the representativeness of the sample.

### Clinical implications

Our findings support routine serum 25(OH)D screening for AA patients, particularly those in urban settings, where deficiency is more pronounced. Given the safety profile, supplementation may serve as a valuable adjuvant therapy, though randomized trials are needed to confirm therapeutic benefits.

## Conclusion

This study confirms that serum 25(OH)D levels are significantly lower in patients with Alopecia Areata compared to matched healthy controls in a North Indian tertiary care setting. The association is notably pronounced in urban populations, likely reflecting environmental and lifestyle determinants. While serum 25-hydroxyvitamin D [25(OH)D] levels did not correlate with disease severity in this predominantly mild cohort, the high odds of deficiency among cases suggest that serum 25-hydroxyvitamin D [25(OH)D] plays a role in disease susceptibility. These findings support consideration of serum 25-hydroxyvitamin D [25(OH)D] status evaluation in patients with Alopecia Areata, particularly in urban settings.

## Data Availability

The data supporting the findings of this study are available from the corresponding author upon reasonable request.
